# Swimming against the tide: A Canadian qualitative study examining the implementation of a province-wide public health initiative to address health equity

**DOI:** 10.1186/s12939-016-0419-4

**Published:** 2016-08-19

**Authors:** Charmaine McPherson, Sume Ndumbe-Eyoh, Claire Betker, Dianne Oickle, Nancy Peroff-Johnston

**Affiliations:** 1 School of Nursing, Faculty of Science, St. Francis Xavier University, Box 5000, Antigonish, NS B2G 2W5 Canada; 2 National Collaborating Centre for Determinants of Health, St. Francis Xavier University, Box 5000, Antigonish, NS B2G 2W5 Canada; 3 Population Health and Health Equity, Public Health and Primary Health Care, Manitoba Health, Healthy Living and Seniors, 4th floor, 300 Carlton St, Winnipeg, MB R3B 3M9 Canada; 4 College of Nurses of Ontario, 101 Davenport Road, Toronto, ON M5R 3P1 Canada

**Keywords:** Social determinants of health, Health equity, Public health, Public health nursing, Organizational capacity, Leadership, Case study, Ideological tensions, Competencies, Policy implementation gaps

## Abstract

**Background:**

Effectively addressing the social determinants of health and health equity are critical yet still-emerging areas of public health practice. This is significant for contemporary practice as the egregious impacts of health inequities on health outcomes continue to be revealed. More public health organizations seek to augment internal organizational capacity to address health equity while the evidence base to inform such leadership is in its infancy. The purpose of this paper is to report on findings of a study examining key factors influencing the development and implementation of the social determinants of health public health nurse (SDH-PHN) role in Ontario, Canada.

**Methods:**

A descriptive qualitative case study approach examined the first Canadian province-wide initiative to add SDH-PHNs to each public health unit. Data sources were documents and staff from public health units (i.e., SDH-PHNs, Managers, Directors, Chief Nursing Officers, Medical Officers of Health) as well as external stakeholders. Data were collected through 42 individual interviews and 226 documents. Interview data were analyzed using framework analysis methods; Prior’s approach guided document analysis.

**Results:**

Three themes related to the SDH-PHN role implementation were identified: (1) ‘Swimming against the tide’ to lead change as staff navigated ideological tensions, competency development, and novel collaborations; (2) Shifting organizational practice environments impacted by initial role placement and action to structurally embed health equity priorities; and (3) Bridging policy implementation gaps related to local-provincial implementation and reporting expectations.

**Conclusions:**

This study extends our understanding of the dynamic interplay among leadership, change management, ideological tensions, and local-provincial public health policy impacting health equity agendas. Given that the social determinants of health lie outside public health, collaboration with communities, health partners and non-health partners is essential to public health practice for health equity. The study findings have implications for increasing our knowledge and capacity for effective system-wide intervention towards health equity as a critical strategic priority for public health and for broader public policy and community engagement. Appropriate and effective public health leadership at multiple levels and by multiple actors is tantamount to adequately making inroads for health equity.

**Electronic supplementary material:**

The online version of this article (doi:10.1186/s12939-016-0419-4) contains supplementary material, which is available to authorized users.

## Background

Improving health equity and addressing the social determinants of health are key priorities in public health practice and organizations. The *social determinants of health* (SDH) are “the economic and social conditions that shape the health of individuals, communities, and jurisdictions as a whole” [1]. *Health equity* refers to “the absence of systematic disparities in health, or in the major SDH, between groups with different levels of underlying social advantage or disadvantage…” [2]. Health equity embraces fairness and justice in policy-related issues, such as service access and affordability, housing, and employment [3]. Health equity and SDH are key considerations in improving and maintaining population health and public health organizations are looking to increase their capacity to engage in these areas.

Organizational capacity ensures that public health organizations can adequately develop, implement and evaluate interventions to address complex social determinants of health. In Canada, public health capacity for health equity action is quite varied and evidence-informed interventions are not fully institutionalized [4]. Public health capacity includes both the internal organizational environment as well as the external or community context [5]. Internally, the values, commitment and infrastructure contribute the organization’s ability to take effective action. Similarly, an enabling external environment including a commitment to public policy to promote health equity further influences organizational capacity [5]. Contextual factors, both external (environmental) and internal (organizational), affect the success of equity initiatives [6, 7] and include structural, human, political, and cultural elements. Examples include institutional policies, organizational hierarchy, decision-making processes, and leadership [7].

Public health organizations across Canada are creating health equity specific staff positions as a means of increasing internal organizational capacity [8]. These positions are typically responsible for developing and implementing health equity strategies and interventions with varied levels of decision-making and formal organizational or leadership authority. Leadership in public health is essential to carrying out the functions associated with improving SDH and health equity [4, 9–11]. Leadership ensures that the core concepts of human rights and social justice are central in planning and implementing activities, specifically through a focus on those at the margins of society [12]. Practitioners in the health equity specific staff positions are providing such leadership.

*Leadership* in this discussion is defined as “the process of persuasion or example by which an individual (or leadership team) induces a group to pursue objectives held by the leader or shared by the leader and his or her followers” [13]. The Public Health Agency of Canada identifies leadership as a core competency for public health practice [14]. Leadership can be enacted at the individual, organizational and systemic levels with the effective integration of science, social strategy, political will, and interpersonal skill required to tackle health equity at any of these levels [15]. Canadian public health figures described effective public health leaders as possessing individual competencies such as the essential knowledge, skills, and attitudes required to advocate for health equity; organizational supports, including allocation of funds, human resources, high-quality population health data, and adherence to external policies and standards; and the ability to connect organizational activities with community action, such as partnering/engaging with community organizations [6]. The values, culture and methods of working of the organization are important to understand public health leadership [16] and shape if and how the organizations acts on SDH and health equity. Leadership is demonstrated by the potential to contribute to how other sectors understand SDH and health inequities and knowledge of the political context; leaders can also serve as catalysts for accelerating innovation and strengthening partnerships [16]. Innovative, strategic, and collaborative leadership and action across several structural levels is critical to effect positive change on complex health and public system issues, such as health inequities [17]. Leadership is a critical factor for the significant reallocation of resources and the shifting of priorities that is needed within health organizations in order to tackle the SDH and health equity.

The creation of new health equity positions necessitates a shift within the organization and systems within which they operate. Dissonance may exist between different bureaucratic layers in public health organizations, creating a challenge for health equity practice [18]. As such, expertise in organizational change management is a critical public health skill [19].

### Introduction to the case

As the largest group of health professionals in the Canadian public health system, nurses are well positioned to lead an “increased focus on disease prevention and health promotion, particularly for vulnerable and underserved communities” [20]. This study examined a province-wide public health initiative to enhance local public health capacity to address SDH and health equity. In 2012, the Ontario Ministry of Health and Long-Term Care (MOHLC) capitalized upon an existing health human resource strategy, “the 9000 Nurses Commitment”, to support Ontario public health units (PHUs) to address health inequities and meet the needs of locally identified priority populations. The 9000 Nurses Commitment was a key component of Ontario’s pledge to increase the overall number of full-time equivalent nursing positions in the health care system [21]. Under the commitment, a broad range of health sectors could apply for funding through the Ministry. Sectors were able to create innovative nursing initiatives to address various gaps in the health care system. Through this funding opportunity the MOHLTC Public Health Division was able to secure funding for each of the 36 PHUs on Ontario to hire two new full-time equivalent public health nurse (PHN) positions focused solely on addressing the SDH.

The Public Health Division set funding requirements to recruit nurses with SDH-specific knowledge and expertise to “enhance supports to program and services needs of specific priority populations impacted most negatively by determinants of health” [22]. The SDH-PHNs would focus on health equity and SDH, including an emphasis on populations most affected by inequities to make health equity central to the activities of public health.

Program and service delivery is mandated by public health legislation in Ontario, including the Ontario Public Health Standards (OPHS) [23], mandatory guidelines issued by the Minister of Health and Long-Term Care. The OPHS articulate the minimum requirements of boards of health in the province to implement public health core functions through the delivery of public health programs and services [23]. The OPHS provide for “a broad range of population-based activities designed to promote the health of the population as a whole, and with community partners to reduce health inequities” [23]. These standards state: “addressing determinants of health and reducing health inequities are fundamental to the work of public health in Ontario” [23]. Through activities such as surveillance and epidemiology, the OPHS note the importance of identifying ‘priority populations’ at risk of poor health outcomes, particularly those most negatively affected by health inequities and SDH. Public health units are required to assess local population health needs, and plan and deliver public health programs and services based on the assessment. Thus, program and service delivery varies across Ontario based on population health needs assessed within each jurisdiction, including those populations where health equity issues are identified.

The purpose of this study was to examine the strategy of developing and implementing equity-focused positions to improve public health organizational capacity to act on SDH and advance health equity. The case study was guided by the primary research question: *What factors influenced the development and implementation of the SDH-PHN initiative in public health?* Sub-questions examined more closely the possible contextual conditions related to that policy and included:

- What key supports and barriers existed in developing and implementing the policy?
- Which key elements of public health leadership were crucial for developing and implementing the policy?
- What activities were undertaken by SDH-PHNs?

## Methods

Case study approach [24] with theoretical propositions guided this study. Propositions related to the substantive research question and subquestions were drawn from existing theory and empirical research on health equity, SDH, and public health leadership [15, 25–28]. Critical for this case study was the use of propositions in lieu of a theoretical framework [24] to: (1) direct attention to particular concepts that should be examined within the scope of the study, and (2) support study feasibility by narrowing the relevant evidence in data collection and analysis [24, 29]. Table 1 provides an overview of the study propositions.

**Table 1:** Study theoretical propositions

Proposition 1	Leadership at multiple levels and by multiple actors is essential for public health actions to address social determinants of health and health equity [15, 25]. Hence, this type of leadership is essential for the SDH-PHN initiative.
Proposition 2	Public health leadership for health equity is context-specific [26] and is highly relational in nature [27, 28]. Hence, public health leadership that considers context and relational factors is important for the SDH-PHN initiative.

Case study is used to understand complex social phenomena when a ‘how’ or ‘why’ question is posed about a contemporary set of events over which the investigator has little or no control, and when trying to trace operational links over time [24]. This approach is particularly useful to examine a complex system, such as the implementation of a new initiative across multiple public health units simultaneously in a relatively short period of time [25]. Further, this approach enabled understanding of policy development and implementation at multiple levels: micro- (individual PHN), meso- (organizational), and macro-level (system, provincial) [25, 30] . This mulit-level approach is particularly critical for complex public systems and health systems change [25].

The Case was a **current event** – a provincial MOHLTC public health initiative in Ontario. To date, this was the first known Canadian province to attempt a province-wide public health policy implementation of this nature, making this a ***unique case*** type, which is important in tracing novel policy development and implementation [24]. This case was bound by time (2012 – 2014); organizational parameters (each public health unit, MOHLTC); geographical/place boundaries (province of Ontario, PHU boundaries as outlined in provincial/municipal legislation); and definition of SDH-PHN (established by each public health unit and MOHLTC).

Data sources included people and documents. Purposeful sampling with maximum variation and predefined criteria (see Table 2) was used to seek sample diversity and breadth across staff from Ontario public health units (i.e., SDH-PHNs, Managers, Directors, Chief Nursing Officers, Medical Officers of Health) as well as other stakeholders (e.g., MOHLTC staff). An emailed letter and an information session offered during a SDH-PHN provincial network conference call meeting were used to introduce the project and recruit participants. Interview data were collected between May 2013 and January 2014. In-depth individual semi-structured interviews lasting approximately 1 h were conducted by telephone, digitally recorded and transcribed. The interview guide consisted of 23 questions, such as: Were there important or controversial issues that arose early on in role implementation? How were these issues resolved? Which processes supported early SDH-PHN role implementation? Which processes inhibited it? Describe how you understand leadership for health equity and the social determinants of health occurring in public health. Do you see the SDH-PHN initiative as an example of public health leadership? This guide changed over the course of the scheduled interviews to reflect the developing themes as data collection and analysis proceeded.

**Table 2:** Socio-demographic characteristics of study participants (*n* = 42)

Characteristic (Pre-defined sampling criteria)	No. of participants (*n* = 42)	% of Total
Gender		
Female	37	88 %
Male	5	12 %
Age		
< 40	11	26 %
40-50	14	33 %
> 50	17	41 %
Role^a^		
Public Health Nurse	24	57 %
Other	18	43 %
Years in Profession		
< 10	10	23.8 %
10-19	9	21.4 %
> 20	23	54.8 %
Years in Public Health Practice		
< 5	11	26.2 %
5-10	6	14.3 %
> 10	25	59.5 %
No. of PHUs represented by participants	22/36^b^	61.1 %
No. of PHUs where both SDH-PHNs interviewed	4/22^c^	18 %
No. of PHUs where more than one role interviewed (always included PHN)	11/22	50 %

^a^Other role category includes Chief Nursing Officers, Directors, Managers, Chief Executive Officers, (Associate) Medical Officers of Health, and Ministry of Health and Long-Term Care staff (numbers per role too small to report)

^b^36 = Number of PHUs in Ontario

^c^22 = Number PHUs represented by study participants

Framework Analysis [31] guided analysis of the interview data. This method was developed in the context of applied policy research [32], and is increasingly used in applied health research [33]. Conceptual scaffolding, a particular method within framework analysis, and its five iterative stages and processes was followed: (a) familiarization, (b) identifying a thematic framework, (c) indexing, (d) charting, and (e) mapping and interpretation [31, 33]. CMP developed codes under the thematic framework, which were verified by SNE, CB, and DO. All co-authors except NPJ reviewed the developing codes and themes.

A variety of documents were purposefully sampled using pre-defined criteria of document type (see Table 3). Documents were collected through an informant process whereby key people associated with the SDH-PHN role development and implementation were asked to identify documents that related to the study questions [34]. Documents were retrieved between May 2013 and February 2014. These documents were analyzed within their social setting as situated products to trace patterns of social exchange and the social networks behind them [34]. Particular attention was paid to: (a) content, not their fixed meaning but a situated or referenced meaning; (b) how they were produced; and (c) how they functioned or their use. Each document was systematically analyzed using a framework that included questions such as: Whose perspective was reflected in the document? How did the document function in terms of SDH-PHN role implementation events and processes?

**Table 3:** Characteristics of study documents (*n* = 226)

Document Type	Examples	Total No. *n* = 226	% of Total
PHU Websites (#1.1 - 1.57)	PHU homepage SDH or Health Equity specific initiative	57	25.2 %
Policy and Planning Documents (#2.1 - 2.95)	Health Unit Strategic Plan SDH-PHN Report to Ministry	95	42 %
Programming Materials (#3.1 - 3.28)	Pamphlet Video	28	12.4 %
Other Communication Materials (#4.1 - 4.46)	Presentation Facebook® page Videos	46	20.4 %

Rigour was assessed through trustworthiness criteria that included assessments of credibility, transferability, dependability, and confirmability [35]. Consistent with a case study approach [24], a chain of evidence was systematically established during data analysis and interpretation, including consistent testing against the study propositions. There was a deliberate focus on divergent patterns, negative instances, alternative themes, and rival explanations [24].

## Results

A sample of 42 participants (Table 2) and 226 documents (Table 3) was achieved. In this paper we report the themes that relate most strongly to the supports/barriers and key leadership elements associated with implementation. The final subquestion regarding SDH-PHN activities is reported through a link in this paper to a data table. For further details on all results we refer the reader to the comprehensive study report [36]. Table 4 provides an overview of the three themes described herein, with supporting quotes identified by participant number (P#) and document number.

**Table 4:** Study themes and subthemes

Theme 1: ‘Swimming against the tide’ to lead change	Theme 2: Shifting organizational practice environments	Theme 3: Bridging policy implementation gaps
• Ideological tensions • Essential competency development • Novel access and engagement with external partners	• Strategic role placement • Structurally embedding health equity	• Gap between public health standards and public health practice environments • Gap between flexible intention and implementation • Gap in reporting processes –‘walking the social justice talk’

### Theme 1: ‘Swimming against the tide’ to lead change

The creation of SDH-focused nurse positions across Ontario PHUs represented a change at the system (provincial) and organizational (local PHU) level. This challenged organizations in several different ways as they implemented the new role because they were ‘swimming against the tide,’ or working against the structural and practice norms in many ways to lead change.

As largely front-line staff, SDH-PHNs had to work within their existing PHU organizational hierarchies. This affected the influence they had on organizational change processes, as indicated by this participant:

We are battling the system all the way. I know what needs to be done, but we cannot get people [unit management and staff] to bend…there is frustration and I feel helpless against the system. I know that I am a better nurse because of this [initiative], but it is not acknowledged. I am very discouraged. I am thinking more broadly about health equity, but nobody wants to acknowledge that. It’s an ‘old boys club’ in public health (P2).

As indicated in Table 4, three subthemes were identified under this theme.

#### Ideological tensions

Data supported the claim that some PHUs had already begun to address SDH in their programs prior to the creation of the SDH-PHN roles, but the degree of programming varied across PHUs.

For many PHUs, developing and implementing the new SDH-PHN position brought forth ideological tensions regarding the role of health equity in public health practice. Many participants reported that the introduction of the SDH-PHN role encouraged their PHUs to shift their approach to health care from largely biomedical and behavioural (e.g., a focus on lifestyle choices) towards acknowledging and acting on the social conditions that affect health and improve health equity (e.g., a focus on social exclusion, early childhood development, income, and education), as outlined in the OPHS [23]. The tension created by this ideological and paradigmatic shift was most apparent in PHUs where the senior leaders’ values and ideologies were not already oriented towards social justice. Participants described this tension in trying to integrate health equity into traditional public health practice:

Many people do not believe in it [health equity work in public health] because it is not so well defined. People are not so overly engaged with the role and they don’t really buy into it. They say ‘What’s the point? Where are we going with this? (P39)

We’ve got a long way to go in public health. The system is not prepared to ….meet the SDH; this needs political will and service providers’ will, not putting people in our traditional public health box (P37).

Participants described tension between colleagues in many PHUs, describing the stressful professional position they were placed in as they launched the new, relatively undefined SDH-PHN roles. Some participants believed this work was already being done and questioned whether SDH-PHNs would now be SDH-health equity experts in the PHU, which had implications for their own competencies and roles. Data indicated that some believed this new initiative was duplication, as identified in this excerpt:

Internally there wasn’t initially great support for this concept. I think initially some of the senior team was thinking well everything we do is SDH, so we’ll just do more of what we’re already doing, versus really wanting to carve this out as separate work. And so there wasn’t widespread commitment across the board early on (P35).

Furthermore, some SDH-PHNs reported receiving clear messages from colleagues that addressing SDH was intractable and beyond the scope of public health practice. However, for some units, the new SDH-PHN role validated health equity work that was already underway, as described by this participant:

Everybody has their own take on the new SDH initiative…some say that it has helped bring practice together – for us it adds to the breadth and legitimacy of our work (P20).

When faced with structural and process barriers in their PHUs, nurses leveraged the OPHS [23] as rationale for program and organizational change and to gain support for SDH work across their organizations. Over time an ideological shift occurred within their units:

We are building internal capacity for the work, staff seem to be soaking it up, the level of awareness is increasing and I’m seeing a paradigm shift (P12).

#### Essential competency development

Participants indicated there was limited direction or evidence to guide their practice regarding health equity since this was an emerging area within public health. The SDH-PHNs and their PHUs spent considerable time at the outset planning the scope of the role including identifying essential competency needs. The SDH-PHNs went through considerable knowledge and skills competency development as they evolved in their roles. This participant described the new role learning curve:

[There is] not a lot of solid evidence in this area about what worked and we needed validation. It was stressful when we started because of the knowledge gap. …. I think I was very naïve and did not anticipate how the role would evolve. There was a definite learning curve for the first 3–6 months (P20).

This was the experience for more senior PHNs who transitioned into the new SDH role as well. However, SDH-PHNs with Masters-level education required less knowledge and skill development and thus moved into the new role more quickly and with more ease.

To address knowledge and competency gaps, SDH-PHNs and their organizations looked to other PHUs that were further ahead in their implementation as examples. Researchers, academic experts, and provincial and national public organizations such as the MOHLTC, Public Health Ontario, and the NCCDH were consulted for SDH and health equity competency development.

For many PHUs there was little infrastructure at the outset to support early competency development for these roles, as noted in these interview excerpts:

The key informant interviews internally and in the community took way longer, and the analysis way longer, and we didn’t have a sufficient epidemiology support built in at the beginning (P35).

So I think there’s been a lot of start-up time and it’s been a bit of a problem enhanced because we’ve had to start up two different people (P27).

#### Novel access and engagement with external partners

To move and entrench a health equity agenda in their PHU’s, many SDH-PHNs began to interact and collaborate with external stakeholders in novel and creative ways. This included a broad array of decision-maker access points and new collaborative partnerships, such as local service providers, interest groups, and communities as well as municipal and provincial government staff and elected officials. This theme also includes the development of some regional and provincial communities of practice.

Study data revealed that PHUs recognized intersectoral collaboration and community engagement as essential to their practice. The SDH-PHN positions provided an opportunity for many PHUs to leverage community partnerships and add a public health voice to multisectoral initiatives, as this participant described:

A support was the existing work of committees in the community. This helped us to link externally and we need to do the systems level work about 50 % of the time and then the other 50 % internally building capacity with our front line group, building team champions. (P4)

The SDH-PHNs formed working relationships with various partners and sectors on issues such as strategic plans and policy change related to health equity. Some partners included municipal departments (e.g., welfare and social services, transportation), poverty reduction coalitions, child welfare organizations, community health centres, social planning councils, libraries, and the education sector. What was different was the political nature of the selected partnerships and the particular focus on the SDH. Interview and document data showed how SDH-PHNs supported health equity work at multiple levels and with multiple stakeholders. But for many units, deep and broad-based coordinated efforts for health equity occurred for the first time. Many study documents depicted the stakeholder and system level engagement (for example, Documents #1.17, 2.23, 3.20), which corroborated the experiences described by participants. The following excerpt exemplifies this new and expanded engagement on a political level:

Our Director facilitated us going directly to our Board of Health. Our engagement with our municipal councillors wasn’t part of the initial plan - that involvement grew organically with the councillors who sit on the Board of Health, the influential level – the movers and shakers, we tried to involve them in the work engaging in real upstream^1^ thinking. (P3)

Novel decision-maker access points had implications at the MOHLTC level. Ministry staff were flexible in their approach to this initiative, supporting a broad array of plans, priorities, and interventions. The parameters for the SDH-PHN roles were not prescriptive, were supported by the OPHS [23], and were based on local planning priorities and principles. The following interview excerpt illustrates this support:

The way this [introduction of the SDH-PHN role] was rolled out provincially was a positive thing for us… we had support but not micro level interference…. this legitimized or authorized some of the things that we were already doing…this gave more clear expectations that this is an expected role of public health…takes a strong public health position on the [health equity] issue (P3).

However, some participants indicated that this flexibility relied on local leadership to drive the implementation. Therefore, role implementation was clearly impeded when local senior leadership was absent or non-supportive. The following interview excerpts illustrate this:

The Ministry leaves a lot of latitude…there are pros and cons to this because it is left to the senior leadership team at unit level. We did ok over time but not for others where unit leadership was not supportive (P5).

The way the Ministry did this worked at the local level for us. Management may be better motivated over time if this is part of an accountability agreement. They are very motivated by those agreements to buy in (P6).

This initiative sparked a shift in how many SDH-PHNs worked with their counterparts across the province. Some areas of the province saw the development of regional and a provincial communities of practice for the SDH-PHNs. These networks provided ready access to nursing leaders and resources (e.g., a Wiki site updated regularly by members), as this participant described:

We developed the provincial network of SDH nurses and from that we have a regional group that was extremely supportive. There was nobody to talk to before this. Now we have good information sharing and a safe zone for brainstorming (P32).

While self-organized, practice networks received support from organizations such as the MOHLTC and the NCCDH through financial or in-kind contributions. Tangible support included infrastructure for teleconference meetings, travel subsidies for in-person meetings, and staff time for workshop planning and delivery.

### Theme 2: Shifting organizational practice environments

Study data highlighted the shifting environments within which practice occurred, including changing organizational structures. It was clear that organizational culture and practice environments shaped how work was structured, which activities occurred, and who was involved in these activities. Further, the data directed us to consider the role of modifiable organizational features and, ultimately, leadership in health equity as priority areas.

#### Strategic role placement

Within each PHU, senior leadership (i.e., senior management/administration) made most decisions about the early development of the SDH-PHN role. This included the scope and placement of the role within the organizational structure. Study data revealed a key issue: lack of consistency in how the SDH-PHN role was positioned across PHUs. This affected several key implementation factors such as the decision-making power for the role, level of practice independence, and role acceptance by colleagues. Participants reported that senior leadership made early decisions based on existing PHU work on health equity (e.g., existence of health equity team; SDH and health equity as an organizational priority), requirements in the OPHS [23] and/or by general operational concerns. In PHUs where little or no consideration for SDH and health equity had previously existed, decisions regarding role focus and placement were not optimal and, in time, required operational shifts. This participant described how early decisions about the roles were made:

I don’t think we really had a clear plan for how this was going to roll out. It was largely the director in consultation with our management group where we kind of discussed if these positions would fit within an existing team, if they needed to be pulled out to kind of work laterally across the different programming areas (P24).

In some instances, the SDH-PHN position was placed within specific program areas, while in others the position was strategically designed to work laterally across program areas and across the PHU. In the vast majority of units, there was an evolution over time where SDH-PHN positions were moved from program-specific assignments to cross-organizational positions, acknowledging that the entire organization needed to be transformed to better tackle health equity. Document data (for example, Documents #1.13, 2.4, 4.7) and interview data corroborated the link among organizational structural placement of the SDH-PHN role, level of practice autonomy, and level of influence for organizational health equity change, as highlighted in these excerpts:

Initially we were bogged down when placed in two different programs, needing to learn program roles, new reporting structure and the two SDH-PHNs were separated. We needed to be able to do broader, system-level work and to support each other. (P4)

The role has changed significantly in terms of whom I report to – from when I started to this time. And so my role has changed in terms of hierarchical changes, where I’m placed, and as a result that’s influenced the level of influence in creating change within the organization (P31).

General patterns of role placement and associated early organizational impacts are outlined in Table 5. Although not every PHN experienced all impacts, study data indicated that many experienced most of the identified impacts.

**Table 5:** Organizational impacts of initial role placement

Initial SDH PHN role placement	Early organizational impacts of role placement
*Assigned to cross-organizational positions*	• worked closely with senior leadership • SDH-PHNs were offered an open-door policy and open communication with senior leadership • worked across organizational departments providing capacity building and technical assistance for other staff • easily connected with other staff also working on heath equity • were perceived as SDH/health equity leaders • experienced a planned organizational approach with clear aim and design to build internal capacity • experienced backlash from colleagues – varied levels of support
*Assigned to specific front-line programs*	• were assigned solely to specific departments • worked within programs or with specific population groups • had less explicit influence on organizational change • experienced SDH/health equity related activities that were more siloed within the organization • tension with time commitments to program delivery and health equity agenda • often difficulty connecting with other SDH-PHN in unit • often difficulty connecting with others working on health equity in agenda • did not experience backlash from colleagues – was not seeking organization-wide support

#### Structurally embedding health equity

Decisions about how SDH-PHNs were positioned within PHUs were linked to the degree that health equity work was already embedded within the structure of the organization. The unanticipated introduction of the SDH-PHN role presented challenges for PHUs as they made decisions about which practices, programs and policies were required to support the initiative. Shifting organizational practice environments included developing organizational processes and documents to structurally embed and support the health equity priority in their organizations.

Nurses worked broadly across their units to develop and implement a collaborative health equity agenda, working with senior leadership in ways that had not been present before the introduction of the role. Notably, some senior leaders also deliberately worked differently than they had previously as they supported the developing health equity efforts, which expanded the opportunity for communication between SDH-PHNs and senior leaders. Examples of adapted mission and vision statements, strategic plans, health equity strategies, workplans, and websites were submitted by participants for analysis as examples of the developing ideological, structural and cultural shifts within their public health practice. These documents were used within current and, for some, newly developed processes and structures (e.g., steering committees, departments) to support the health equity agenda. Participants noted the inclusion of health equity in regular meetings, reports, and other communications, as identified in this excerpt:

At the health unit we redid our strategic plan to include SDH and health equity. We are treated like any other department now. We didn’t think about it before, but we can’t ignore it now. This has really taken our unit out of its comfort zone. The whole municipality is recognizing this work (P2).

Data revealed that the SDH-PHN roles supported the broad-based work of all PHU staff on SDH and health equity. This had implications for how the SDH-PHNs worked with others in their units and for cross-unit competency development, as reflected upon by this participant:

We are building internal capacity for the work….We make time to talk about the SDH and health equity at every staff meeting, so it is seen as always a learning opportunity now (P12).

As new SDH-PHN positions were filled, and depending on the position’s organizational placement and senior administrative support, some SDH-PHNs began to be involved in determining overall organizational activity around health equity. Participants described support from middle and senior management, encouraging autonomous practice for the SDH-PHNs and deliberately building the position into the organizational structure so they could have a critical role in the planning process. Planning autonomy was more apparent in units where senior management supported the role. Some PHUs developed health equity teams and/or advisory committees to provide leadership and guidance for the organization’s initiatives. Some of these structural supports predated the SDH-PHN position initiative; however, in many organizations these supportive structures were created after the initiative was implemented, as identified in this passage:

The new reporting structure has shifted this role to the senior leadership team with our [senior health equity working group] and working across the unit has made a huge difference. I pushed the health equity agenda – this was greater than working through two program teams. (P4)

Advisory bodies or working groups related to SDH and health equity typically had broad organizational representation, providing a direct link to senior decision-makers and multidisciplinary staff who supported the SDH-PHNs and SDH work across the organization. Senior leaders demonstrated their support for SDH activities and created legitimacy for the ongoing work, as noted by this participant:

The structure and culture of the health unit is very important. You need a Medical Officer of Health who is in tune with the staff, where nurses are able to approach them, no filtering needed. When the MOH chairs the committee it gives clout, power. (P4)

However, some participants clearly indicated that a lack of senior and middle management support was detrimental for the role and for the health equity agenda, and so management became a focus for change. For example:

There is middle management opposition. They say they already know this [i.e., health equity work] and are already doing this. We need to win them over with evidence and best practice. We see them have their ‘ah-ha’ moments though (P3).

Support for this work was evident in document data such as the Ontario Public Health Standards PHS (OPHS) [23] and the Ontario Public Health Organizational Standards [37]. These documents mandate boards of health for PHUs to incorporate strategies addressing health equity into their strategic and program plans. Specifically, PHUs were to develop a strategic plan that “describes how equity issues will be addressed in the delivery and outcomes of programs and services” [37]. The OPHS were released in 2008 and, at the time, the work of some PHUs on health equity was well ahead of others and well ahead of the implementation of the province-wide SDH-PHN initiative. Participants recognized that health equity work was already embedded in the OPHS, although many commented on the lack of practice direction and the mismatch with the current public health structural practice environment, as described by this participant:

Adding health equity was required…it is embedded in our public health standards anyway. Where we are left though is it just feels like square peg in a round hole compared to how we traditionally work. We are in a huge panic to get indicators, outcomes – how do we measure success for this? One size is not fitting all across the province (P3).

### Theme 3: Bridging policy implementation gaps

Participants reported that a gap between policy visions at one level and actual implementation at another inhibited the initiative. These policy implementation gaps, such as lack of implementation plans at the local unit level, were considered by participants as barriers to the long-term sustainability of the SDH-PHN initiative and for other future initiatives. Many SDH-PHNs worked to bridge these gaps.

#### Gap between public health standards and public health practice environments

Support for the SDH and health equity work was evident in document data such as the Ontario Public Health Standards (OPHS) [23] and the Ontario Public Health Organizational Standards [37]. These documents mandate boards of health for PHUs to incorporate strategies addressing health equity into their strategic and program plans. Specifically, PHUs were to develop a strategic plan that “describes how equity issues will be addressed in the delivery and outcomes of programs and services” [37]. The OPHS were released in 2008 and, at the time, the work of some PHUs on health equity was well ahead of others and well ahead of the implementation of the province-wide SDH-PHN initiative. Participants recognized that health equity work was already embedded in the OPHS, although many commented on the lack of practice direction and the mismatch with the current public health structural practice environment, as described by this participant:

Adding health equity was required…it is embedded in our public health standards anyway. Where we are left though is it just feels like square peg in a round hole compared to how we traditionally work. We are in a huge panic to get indicators, outcomes – how do we measure success for this? One size is not fitting all across the province (P3).

#### Gap between flexible intention and implementation

Document data revealed wide variation between local level interpretation, planning, and implementation of the SDH initiative and the Ministry’s vision for *locally-informed* planning, as per the OPHS [23] (e.g., Documents # 2.1, 2.13, 2.23). Some PHUs embraced the new SDH-PHN positions to address health equity issues, coupled with much effort and internal change. However, other PHUs viewed the positions as merely the addition of more full-time equivalent PHN positions and, as a result, added them to existing programs without a clearly enhanced or expanded broader plan, strategic priority, or structural changes around health equity.

Some participants described how the broad guidelines developed at the provincial level created a gap between the Ministry’s vision and the actual implementation at the local health unit level. Many participants perceived this as a lack of direction or clarity, not as an intentional shift in approach to increase Ministry flexibility and movement to more locally driven planning as outlined in the OPHS [23]. Notably, this perception of limited guidance from the Ministry was primarily an issue for those units that were not already advanced in health equity work, resulting in a disconnect between the intent for locally-driven action and the actual local implementation experience. These interview passages highlight this issue:

The role implementation was controversial in the beginning…there was lack of role clarity from the province and in the health unit itself, and lack of clear communications internally. This led to many assumptions about what we should or should not be doing. Early on this devalued the role. This shifted over time – there is less role confusion as health equity has become a strategic direction, so who owns what, whose portfolio this is in is clearer. (P5)

There was too much flexibility. Units are all over the map with this…they need goals, need to go back and measure what we really did; need more of a sense of what they wanted out of the project (P37).

Alternatively, some PHUs embraced the flexibility of the initiative and welcomed the new positions as validating and enhancing their existing work. They tailored the new positions to their local PHU needs, stage of development, and capacity to address SDH and health equity. Some participants noted that even more flexibility pertaining to the staff (i.e., that the positions not be limited to PHNs) would have been welcome. At these PHUs, the local health equity agenda matched the provincial policy (i.e., there was no identifiable policy implementation gap), as these participants articulated:

Fortunately, for our health unit, the SDH were a priority [before the SDH-PHN role was introduced], the SDH positions were the real impetus to move this along. This has really put SDH on the agenda and made health equity a priority. There is a trickle-down effect into programs and other professions. There is momentum as it is now a priority. The Ministry flexibility was key from the beginning (P20).

The parameters were broad. I think this is always very good so each health unit can take it and customize it. Some health units may have struggled because they needed more direction. The Ministry put it out to the field and let us work with it (P21).

#### Gap in reporting processes – ‘walking the social justice talk’

A gap was identified between provincial policy in terms of reporting expectations and local practice. This may be considered a gap between expected behaviour of public health practitioners that would be consistent with social justice and equity-informed practice and their actual practice – or ‘walking the social justice talk.’ Many SDH-PHNs reported having neither input into the regular report to the Ministry, nor access to the final submitted reports, despite the Ministry reporting requirements (Document # 2.45). The Ministry required that both front-line staff in the SDH-PHN role and their direct management reports contribute to these reports. The intention was that both could provide detail and nuanced information that might not be captured by senior leadership alone since they may not be familiar with day-to-day operations of the role. A gap formed between what the nurses were doing in practice and the communication of their work within the provincial accountability structure. This situation was clearly described by several participants, as illustrated in this excerpt:

My manager did the Ministry report for year 2 without my input. I asked if I could see it because other SDH-PHNs were talking about it. I didn’t even see the year 1 report (P37).

Alternatively, PHUs that were already demonstrating leadership in health equity modeled social justice and equity-informed behaviour through engagement at the unit level. As a result, the SDH-PHNs nurses either co-authored or authored (with collaborative input) their PHU reports to the Ministry, as described in this excerpt:

We wrote our Ministry reports from day 1. Who else would know the detail required for this? We collaborated with other staff and senior leadership to do this of course (P8).

Support for this work was evident in document data such as the Ontario Public Health Standards PHS (OPHS) [23] and the Ontario Public Health Organizational Standards [37]. These documents mandate boards of health for PHUs to incorporate strategies addressing health equity into their strategic and program plans. Specifically, PHUs were to develop a strategic plan that “describes how equity issues will be addressed in the delivery and outcomes of programs and services” [37]. The OPHS were released in 2008 and, at the time, the work of some PHUs on health equity was well ahead of others and well ahead of the implementation of the province-wide SDH-PHN initiative. Participants recognized that health equity work was already embedded in the OPHS, although many commented on the lack of practice direction and the mismatch with the current public health structural practice environment, as described by this participant:

Adding health equity was required…it is embedded in our public health standards anyway. Where we are left though is it just feels like square peg in a round hole compared to how we traditionally work. We are in a huge panic to get indicators, outcomes – how do we measure success for this? One size is not fitting all across the province (P3).

### Leadership

This study examined the key elements of public health leadership that were crucial for developing and implementing the SDH-PHN initiative. We explored leadership at the individual, organizational (PHU), and systems levels. Some leadership issues were integrated into the earlier presented themes as there is not a clear distinction between many of the aspects of the presented themes and the leadership question. We highlight particular key aspects here.

#### Individual level leadership

Participants consistently indicated that leadership was foundational to action on the SDH and health equity, as expressed by this participant:

I think it’s [i.e., leadership] the cornerstone of what we do, and if you look at the core competencies for public health that were articulated by Public Health Agency of Canada, …it’s foundational to all the work that we do, looking at encompassing the values of public health, like equity, social justice, community participation and the whole determinants of health framework, that’s where all of that comes from, and so I think that offered a very solid foundation for leadership (P33).

Many participants reported that PHU leadership consistently adapted to the emerging SDH-PHN role and the associated work. These leaders, described as “strong,” “engaged,” “trusting,” and “risk-taking,” enabled the SDH-PHNs to reorganize as they learned and grew. Further, participants described leadership as “forward-looking,” “authentic,” and “critically introspective.” These excerpts highlight the risk-taking and forward-thinking aspects of leadership:

I think it’s the push…leadership would be out there pushing the edge and the envelope, not just doing things just when it’s been made easy for you in a sense (P35).

Leadership would begin with acknowledging where we’re not doing a good job, and then be willing to engage in an authentic discussion of where we might not be doing a good job and be willing to actually take some responsibility for that…knowing that’s going to mean saying no to some things (P40).

#### Organizational/public health unit level leadership

Many individual SDH-PHNs emerged as leaders within their organizations. At the PHU level of implementation, SDH-PHN leadership was most often described as “doing the work” and fulfilling the requirements of the role. These nurses guided their colleagues and organizations, worked with community and intersectoral partners, and were passionately involved in advocacy and innovative public policy change. In particular, the SDH-PHNs were considered leaders when they practiced in ways that championed health equity, as described in this excerpt:

I think they demonstrate leadership because they’ve become sort of like champions, they are very knowledgeable, and they are very connected in the community around the specific issues. They’re really passionate about it and they want to move the agenda forward (P27).

Formal leaders who acted as champions at the governance and senior management levels enabled action and were identified as essential to health equity activities. These leaders embedded SDH and health equity in the overall direction of the organization in a deliberate and purposeful manner. This action took various forms including making health equity part of strategic priorities, mission statements and programs. Strong leadership empowered PHUs to take a nuanced approach to their work, establishing public health’s unique role in improving SDH. These leaders also considered work on SDH and health equity a natural part of public health, as exemplified in this passage:

I think public health always wrestles with how much we should be influencing or can be influencing the SDH. We recognize that the foundation that everything in public health is related to the SDH… within an organization having leadership understand how important SDH are, are really important to move it forward and make it more integrated in how all your programs are delivered (P27).

At the organizational level, leadership was demonstrated through a range of practices and activities within PHUs (e.g., raising awareness, supporting capacity building within the organization), through direct service delivery (e.g., redesigning public health programs, developing strategic or programmatic guidance and direction, and identifying and working with priority populations affected by inequities).

#### Systems level leadership

Systems level leadership was demonstrated through collaborative policy action (e.g., interorganizational and cross-sector collaboration, naming health equity issues publically outside of the organizations, targeted policy advocacy). Participants provided examples of local PHUs and provincial and national organizations they considered health equity leaders. These PHUs acted both internally and externally on the SDH and health equity, as indicated by this participant:

I know that public health plays a pivotal role in providing some of this leadership across our catchment area. And it is a natural fit for public health, it really is some of the core of what we do and why public health exists in the first place, the foundation of what it is. So for us it’s not only how we deliver our services, but how we work with our community partners and some of the initiatives that we participate in even though we might not be a direct service provider we help to facilitate or support in order to address some of the health equity issues within our community (P26).

Participants preferred leadership that was distributed across the organization, in other sectors, and within the community, rather than directed through a particular program population. As discussed under theme 1, health equity was not always widely accepted as the ‘business’ of public health, which impacted the ability of SDH-PHNs and their organizations to move forward on this issue internally. Theme 3 examined how local perception of provincial leadership varied. Some saw provincial funding as a clear sign of Ministry leadership and as a means to further action on directions indicated in the public health standards. Others had mixed feelings and some perceived the limited guidance and action at the provincial level as a lack of leadership, as this participant indicated:

No I don’t [believe that was provincial level leadership]. Well in what ways is it leadership? ….are two extra nurses on SDH is going to make any difference? (P34)

Table 6 offers examples of supports and barriers identified throughout data analysis and interpretation. Identifying the tangible supports and barriers is intended to augment the presented themes and leadership sections, which may help guide public health organizations in developing and implementing SDH and health equity roles.

**Table 6:** Supports and barriers in developing and implementing the SDH-PHN role

SUPPORTS
• Health equity already integrated into the OPHS and the Ontario Public Health Organizational Standards • Availability of MOHLTC funding and ongoing support for SDH-PHN positions (e.g., support for provincial SDH-PHN network teleconference line) • Availability of skilled and knowledgeable SDH-PHNs • PHNs with Masters-level education; PHNs with previous health equity experience • Investing in training SDH PHNs and other staff • Investing time and resources at outset for health equity learning • Access to expertise, both internally and externally, supported competency development and knowledge acquisition • Strong leadership at governance and senior management levels enabled action • Development or expansion of internal organizational structures such as working groups, steering committees or departments provided a centralized position for SDH/health equity priorities within the organization • Structurally embedding health equity concept and role: in planning documents (e.g., strategic plans, health unit vision statements) and in processes (e.g., steering committee, reporting member of management and planning committees) • A flexible and adaptive approach allowed organizations to learn and adapt to changing needs and requirements • Open communication and information flow facilitated SDH-PHN role by providing access to staff in all programs • SDH-PHN involvement in organizational decision-making regarding the priority area was necessary to implement health equity activities • Breaking down siloes as part of the planning process facilitated cross-organizational networking and shifts in organizational structure • Networking among nurses and other stakeholders assisted SDH-PHNs and their organizations to build relationships with those doing similar work, to learn from the experiences of others, and to access information and knowledge • Nurses demonstrated leadership in self-organizing knowledge exchange and network development opportunities (e.g., regular meeting of SDH-PHNs across province) • Ongoing support from provincial government (i.e., MOHLTC) and national organization (i.e., NCCDH) for SDH-PHN provincial network • Partnering with external health and non-health organizations, networks and policy-makers allowed PHUs to identify a focus for the SDH-PHNs and to be responsive to the local context • SDH-PHNs given access to policy makers, where they often exercised policy development and advocacy roles • PHUs with a history of action on SDH and health equity implemented the roles more easily, as these were viewed to support and enhance ongoing work
BARRIERS
• Ideological tension between biomedical and behavioural/lifestyle paradigm and shift to public health practice to address health equity • Systemic organizational oppression and power dynamics that are stifling for PHN roles • Some public health staff completed their professional education before health equity concepts were integrated into entry to practice professional programs. This contributed to a tension in terms of the required knowledge, skills and values required to support integration of health equity into public health practice • Public health staff belief that influencing social systems in the service of equity was extremely difficult and too broad-based • In units without pre-existing health equity programs, questioning of the evidence base with which to approach health equity work • Perceived lack of clarity regarding provincial expectations was a stumbling block especially for PHUs in the early phases of health equity action. Despite the existence of the OPHS, data highlighted a lack of clear guidance for early implementation • Staff or management transitions and instability related to the position created uncertainty about continuity of the work and support at different levels of the organization. Some staff changes in the SDH-PHN role were the result of normal turnover while others were related to the skills and competencies of the individual in that position. • Limited knowledge of evidence to support local public health action meant that health units spent time trying to identify what to do and how to do it. This was often pre-empted by the need to make the case for health equity action where this was considered an extra responsibility. • Lack of internal coordination within some PHUs impeded role implementation; role could have been better linked to other related work in PHU to move out of siloes • Discipline-specific funding led to some internal frustration, tension and feelings of exclusion early on. While data showed that staff understood why the funding was PHN-specific, some PHUs would have preferred to focus on the skills and competencies required and have the ability to draw from the multidisciplinary perspectives that are often required for SDH/health equity work.

An additional table file shows examples of activities implemented by SDH-PHNs (see Additional file 1). These activities were elements embedded in the data forming study themes and subthemes. These activities highlight specific examples reported by SDH-PHN participants, not an analysis of effectiveness at various levels.

## Discussion

This study explored the development and implementation of the SDH-PHN initiative in Ontario. The study findings identify three essential areas to be considered by leaders and practitioners in contemporary public health practice to support strategic roles and policy regarding health equity. The implementation of specialized SDH-PHN positions across Ontario PHUs required a tangible shift in public health practice. Our findings identified the importance of forging ahead to lead the health equity agenda in the face of traditional public health ideological, structural, and collaborative practice constraints; of paying particular attention to early role scoping and to purposefully embed health equity in the structure of PHUs; and of identifying and shifting known policy implementation gaps. Particular leadership factors at play in the SDH-PHN initiative were also surfaced. Significantly, and consistent with study proposition 1 (see Table 1), the need for active engagement at multiple levels and by multiple actors was identified as key in enhancing the success of the initiative.

The study findings yield important and novel insights for developing and implementing public health SDH/health equity specialist roles broadly, especially in terms of enhancing traditional PHN practitioner roles, the sharing of power within the public health setting, and organizational readiness for equity work. A key issue identified in this study was how crucial the organizational culture was in acting as a support or barrier for SDH-PHN role development and implementation. Organizational culture includes those basic values, assumptions and behaviours that influence the functioning of the organization. These are often taken for granted and represent a powerful force affecting the activities of an organization [38]. Supporting proposition 2 (see Table 1), the context of the organizational culture emerged as a key factor supporting a PHU’s ultimate ability and motivation to advance a health equity agenda through the SDH-PHN role. Our findings are similar to recent Ontario research highlighting the influence of the Medical Officer of Health and staff ideology and organizational structures on public health action on health equity [18]. The notion of organizational culture and embracing the health equity challenge, and more fundamentally, prioritizing health equity were profiled in our findings.

This study examined the SDH-PHN role within each PHU organizational context. Organizational culture affected the ability of the SDH-PHNs to develop their roles and the health equity priority. The value (or lack thereof) placed on nursing roles in some PHUs, compounded with the struggle to embrace health equity as an integral part of contemporary public health practice, influenced the role development and implementation. This inevitably had an impact on health equity aims. Organizational oppression and power was a barrier in some units, which slowed progress toward a unit-wide health equity agenda. Organizations can be a site of empowerment or oppression, and power within organizations limits or enhances the actions and capacities of the professionals within the system [16]. The structure of the work environment and access to power and opportunity influences the attitudes and behaviours of individuals within organizations. Historically, power and empowerment tensions have existed for the nursing profession. Nurses may be more reluctant than most to discuss power because 90 % of all nurses in Canada are women [39], and women have traditionally not been socialized to exert power [40]. Definitions of power can include the ability to get things done and to mobilize resources [41], so negative structural power issues in clinical practice can have major implications for effective practice and client outcomes. Empowerment for nurses may consist of three components: a workplace that has the requisite structures to promote empowerment; a psychological belief in one’s ability to be empowered; and acknowledgement that there is power in the relationships and caring that nurses provide [41]. In organizations that demonstrated empowering attributes, the SDH-PHNs were better positioned to grow the new role and contribute to, and in many instances to lead, an enhanced health equity agenda. These organizations structured the opportunity for SDH-PHNs to increase their knowledge and skills, access and mobilize resources, and develop and implement plans to make equity practice change. These findings are consistent with other public health research that examines Canadian PHNs’ practice from a management perspective [42–44]. Constraining structures, operations and governance, in addition to insufficient infrastructure support are part of the unique “day-to-day contested realities of public health and PHN practice” [44].

The nature of PHU senior leadership styles was crucial in this investigation. Annett [16] noted the importance of understanding the values, culture, and methods of working of the home organization; the potential to contribute to the understanding of determinants of health and health inequities in other sectors; and knowledge of the political context. Leadership roles in public health can serve as catalysts for accelerating innovation and strengthening partnerships. Public health leadership also requires being “attentive to social conscience and scientific intent” [16].

Leadership issues influenced the impact of organizational culture on developing and implementing the new positions—senior leaders had the power to structure the role for effective implementation. This is part of proposition 2 (see Table 1) and extends our understanding regarding the context-specific [26] and highly relational nature of public health practice for health equity [27, 28] . We know that leadership style and organizational empowerment are intricately linked [45]. Organizational culture and management and leadership practices have been identified as being supportive to successful public health nursing practice [42, 43]. When effective leadership permeates an organization, its members feel empowered and motivated to be effective in their roles. Without this type of leadership, the organization may miss opportunities to integrate new methods of solving problems or learning [38]. Managers need to understand the role of PHNs who work for them and make it possible for nurses to use the full scope of their competencies [43]. Local organizational culture that supports PHNs to best practice the full scope of their competencies includes effective leadership that values diverse public health roles, demonstrating respect, trust, and support for PHNs [42]. Underwood and colleagues [43] and Meagher-Stewart and colleagues [42] underscored particular organizational attributes in public health that are relevant to community health nursing capacity in Canada. Attributes that contribute to this focus on work processes and relationships include shared vision and goals, partnerships and collaboration, creativity and responsiveness, learning, and information sharing. Consistent with the findings in this study, others have identified the benefits of having the time to build partnerships, respond to new program opportunities, and pursue ongoing professional development [27, 28, 42].

Senior leadership decision-making was central to a key finding indicating that it is valuable to design and place the SDH-PHN roles cross-organization at the outset. Supporting study proposition 1 (see Table 1), our findings expand this understanding by identifying the multiple actors and levels at which they work towards health equity, including cross-organization SDH-PHNs collaborating with senior leadership and their organizational colleagues. Also supporting study proposition 2 (see Table 1), these findings expand our understanding by identifying the context-specific aspect of building a health equity agenda. In this study, we saw the impact of early contextual decisions on the role implementation over time. This positions health equity as a priority issue for the organization, increases staff competencies around health equity, and provides support for integrating SDH and health equity *across* the organization. In contrast, assigning the positions to specific program areas at the outset, where they tended to be submerged in day-to-day front-line and often individual service provision, albeit with priority populations, resulted in an overall lack of visibility of equity work at the cross-organizational level. This was especially detrimental in organizations with little or no broader health equity strategy at the initiative outset. The organization-wide option offered the SDH-PHNs ‘permission’ to do the work and enhanced the power base from which they operated. This built the health equity agenda more quickly and strategically within the organization, including peer engagement and professional growth regarding health equity practice. The SDH-PHNs were supported in building an evidence-based equity plan that was cross-disciplinary in nature, which is essential to building healthy public policy [46].

The study findings indicated the existence of a policy implementation gap between public health standards and actual health equity planning and action within some PHUs. However, many PHUs used the opportunity to develop the SDH-PHN positions to increase their strategic focus on health equity—far beyond where they were working before the introduction of the position. It was clear that the SDH-PHN initiative had a significant positive impact on the capacity of many PHUs to advance strategically focused health equity plans. However, despite the nature of the OPHS [23] and the Ontario Public Health Organizational Standards [37] as directives to address health inequity and SDH, many at the local PHU level perceived that provincial guidance on these issues was lacking. This contributed to frustration at the local level, notably for PHUs at the early stages of health equity action. What was originally intended as leeway to support local, evidence-based decision-making, which is fundamental to contemporary public health practice, was interpreted by some as lack of direction by provincial public health policy makers. This suggests that standards alone are not sufficient to shift practice at the local level, especially considering the stronghold of traditional public health programming.

The study findings highlight the need to make public health structural and cultural organizational changes to intentionally make room for a health equity agenda. There is a growing recognition that changes to health care systems and organizations require integrated action, with each system area incrementally reinforcing and developing other interdependent areas [42]. The results of this study highlight areas for public health organizational development and offer recommendations for supporting effective public health practice to support health equity and to avoid policy implementation gaps. Table 7 offers recommendations that further address the identified policy implementation gaps.

**Table 7:** Recommendations based on the research findings

FOCUS	RECOMMENDATION
Policy	• Local involvement in policy development at the provincial level may help reduce policy implementation gaps. • Increased dialogue and communication about requirements and expectations prior to role creation and funding could enhance clarity across the public health system. • Ongoing engagement through discussion across multiple system levels can also help avoid and address major policy implementation gaps. • Providing guidance to PHUs on how to implement health equity mandates while maintaining flexibility for local adaptation will support implementation. • Clear accountability measures built into accountability agreements can help ensure that positions are used to meet the intended mandate of increasing organizational health equity capacity. • Ongoing feedback mechanisms between provincial and local stakeholders can help ensure that local public health organizations have the support needed to fully implement health equity positions and related activities. • Taking action on the SDH and health equity requires a multidisciplinary approach. Human resource initiatives that draw from a range of disciplines will benefit from diverse skills and perspectives. • Support for knowledge exchange and network development for those in similar roles enhances information sharing and joint planning, and will amplify gains across organizations.
Practice	• Including health equity considerations in program planning and delivery supports public health unit staff, including SDH-PHNs, to consistently and explicitly work to address SDH and health equity. This would help to address doubts about the role of public health in addressing SDH/health equity, alleviate tension in the practice environment, and demonstrate organizational and leadership support for the work. It would also underline the need to shift approaches, from a largely behavioural and biomedical to a SDH and health equity focus. • Clearly defined responsibilities for health equity positions (built into accountability agreements that draw explicit links between provincial mandates and locally planned actions) would minimize the disconnect between provincial plan intentions and local health unit interpretations. • Organizations seeking to better address the SDH and health equity must look internally and align their workplace values, culture, and practices with equity and social justice. By doing so, they create an environment for professionals to develop a reflexive public health practice. • Public health organizations that 1) develop and promote cultural attributes (such as a shared vision, mission, and goals) that prioritize health equity and are understood and valued throughout the organization, and 2) foster a culture of creativity and responsiveness, will support PHNs and other staff to practice the full scope of their competencies. • A supportive learning environment in which there is continued development that enables staff to gain the skills required to be effective in their roles. This means cultivating a healthy organizational culture in public health by: o transforming power relationships within the organization and beyond, o encouraging access to and free flow of information, o supporting innovation and new methods, and o creating an engaged earning environment. • Internal and external activities serve to bolster the work of the organization. Ensuring that internal structures are in place brings public health staff together and helps reduce internal siloes. Given that the SDH lie outside public health, working in collaboration with communities, health partners and non-health partners is an essential part of the health equity role. • Visionary and empowering leadership supports the integration of health equity as part of everyday public health practice. Enhancing these leadership styles will help further organizational action.
Education	• All disciplines in public health must receive continuing education and professional development in addressing SDH and health equity to support the development of knowledge and skills. • Competency development across the organization would allay concerns of being siloed, disperse collegial tension, and position health equity specialist roles within a supportive framework. This allows for “leadership from within” on health equity. • Competencies highlighted in this study include: o knowledge of SDH and health equity o organizational change/development o systems change strategies o program development and evaluation with specific consideration to equity o advocacy o policy development o community engagement o leadership
Research	The critical yet still-emerging area of health equity and addressing SDH would benefit from further research that examines the following: • the relationship between organizational culture (including values and ideology) and an organization’s capacity to work on a health equity agenda • the impact of structurally embedded workplace inequities (e.g., disempowerment of nurses) on health inequity priorities • the activities of SDH-PHN and their influence on their respective organization’s capacity to address health equity work • the disciplines and public health professionals best positioned to effectively advance the health equity agenda and how best to prepare/educate practitioners for these roles • the development of similar public health roles in other jurisdictions to strengthen the science behind public health equity work and to increase the strength of the transferability of the findings reported here

Significantly, it was evident throughout the investigation that there are passionate and committed SDH-PHNs and managers who have the capacity to effect and are actively pursuing health equity change. They focused on organizational health equity capacity building, and ultimately on improving health equity outcomes for their communities at large and priority populations specifically.

Findings from this study need to be considered in light of study limitations. The reader should be careful to not generalize from a single case study design, but rather to consider the degree of theoretical transferability and fittingness to other contexts. That some participants were new to the SDH-PHN position was a limitation as they had very little first-hand knowledge of the early role experiences and processes. A major strength of this work was the use of propositions as the theoretical basis for study design, allowing us to draw on multiple theoretical perspectives. The use of Framework Analysis, which was originally developed for public health system research purposes, strengthened the analytical process and the credibility of the findings.

## Conclusions

This study suggests that many public health units benefitted greatly from the investment in the form of SDH-PHNs—the initial funding, vision and ongoing MOHLTC support served as a catalyst to address SDH in some PHUs and enhanced existing health equity work in others. Building on existing research, this study extends our understanding of the dynamic interplay among leadership, change management, differing ideologies, organizational cultures, and interjursidictional policy efforts impacting health equity agendas in public health practice. Our findings give voice to Ontario PHU staff regarding the facilitators and barriers experienced in the implementation of this health equity innovation. Findings showed that PHUs particularly benefitted from the implementation of cross-organizational SDH-PHN positions, structurally embedding health equity as an organizational and system priority that engaged many actors internally and externally. These study findings are important and relevant for contemporary public health practice as the impact of health inequities on population health outcomes continues to come to the fore. Given that the SDH lie outside public health, working in collaboration with communities, health partners and non-health partners is an essential part of the public health role for addressing health equity. Increasing our knowledge and capacity for effective system-wide intervention towards health equity are critical strategic priorities for public health practice, broader public policy, and community engagement. Appropriate and effective public health leadership at multiple levels and by multiple actors is tantamount to adequately make inroads for health equity.

### Recommendations

The findings of this study have implications for policy, practice, education, and research. Table 7 provides recommendations for consideration in these areas.

## Abbreviations

MOHLTC, Ontario Ministry of Health and Long-Term Care; NCCDH, National Collaborating Centre for Determinants of Health; OPHS, Ontario Public Health Standards; PHN, Public health nurse; PHU, Public health unit; SDH, social determinants of health; SDH-PHN, social determinants of health public health nurse.

## Additional file

Additional file 1: Examples of activities implemented by SDH-PHN positions Specific examples reported by SDH-PHN participants classified according to public health roles, components of public health action and level of practice, as interpreted by the authors. AdditionalFile_ExamplesSDHPHNactivities.pdf. (PDF 43 KB)

## Footnotes

1 Upstream refers to acting on (1) the structural determinants of health by implementing policies and practices that shift the distribution of power and resources, and (2) the causes of social disadvantage.
