# Supplementary material for: Swimming against the tide: A Canadian qualitative study examining the implementation of a province-wide public health initiative to address health equity
Source: Int J Equity Health. 2016 Aug 19;15:129. doi: 10.1186/s12939-016-0419-4 (PMC4991018; doi:10.1186/s12939-016-0419-4)
Supplement: Additional file 1: — Examples of activities implemented by SDH-PHN positions Specific examples reported by SDH-PHN participants classified according to public health roles, components of public health action and level of practice, as interpreted by the authors. AdditionalFile_ExamplesSDHPHNactivities.pdf. (PDF 43 KB) [file 12939_2016_419_MOESM1_ESM.docx]

**Examples of activities implemented by SDH-PHN positions**

Specific examples reported by SDH-PHN participants, including initial and current implementation stages. Combining of concepts by authors for simplicity. Classification according to public health roles, components to public health action, and level of practice as interpreted by the authors. This is intended to be a reporting of what was stated, not an analysis of effectiveness at various levels. Individual points may be applied to other public health roles, components to public health action, and practice focus/scope.

| **Public Health Roles** | **Components of Public Health Action** | | | | | | **Practice focus/scope** | | |
| --- | --- | --- | --- | --- | --- | --- | --- | --- | --- |
|  | **Leadership** | **Develop/apply information** | **Education & Awareness** | **Organization & System Dev.** | **Skill Dev.** | **Partnership Dev.** | **Organi- zational** | **Regional** | **Provincial**  **or National** |
| **Assess & report on health** |  | Advocate for RRFSS* module on public awareness of SDH |  |  |  |  | √ |  |  |
|  |  | Review research and work with epidemiologist to understand strategies to support the identified priority population |  |  |  |  | √ |  |  |
| **Modify/Orient public health interventions** | Chair the health equity/ SDH specialist team within health unit | Work in specific roles – e.g., street nurse, deliver direct service to priority populations | Consult with staff on health equity and SDH related topics, program planning | Attend program & department meetings across public health | Support staff with the use of health equity and SDH related tools, framework |  | √ |  |  |
|  |  | Focus on priority populations in specific program area – urban issues, chronic disease, child & reproductive health, school health, HBHC* | Prepare internal communications related to SDH and health to support ongoing communication and knowledge | Develop health unit approach to addressing SDH , policy, agency operational and program plans |  |  | √ |  |  |
|  |  | Conduct SDH training for other staff | Develop online prenatal education module | Divide SDH-PHN positions among programs in smaller increments – e.g., 0.1 FTE |  |  | √ |  |  |
|  |  |  | Develop website for agency to address SDH | Develop agency-wide SDH and health equity tools – equity impact assessment tool, framework, guidance document, decision making tools, process to identify priority populations |  |  | √ | √ |  |
| **Engage in community & intersectoral collaboration** |  |  | Knowledge broker to other health care providers – community health centres, primary care practitioners |  | Organize workshops and training on SDH for community partners | Link with external agencies that work with SDH affected clients – e.g., newcomer agencies, housing |  | √ |  |
|  |  |  |  |  |  | Work with stakeholders to understand how SDH impact health |  | √ |  |
|  |  |  |  |  |  | Outreach to urban Aboriginal area and organizations |  | √ |  |
| **Leadership; policy analysis, development, & advocacy** |  |  |  |  | Participate in local coalitions linked with sdh-related factors | Involvement with Ontario provincial SDH-PHN network and alpha-OPHA health equity work group |  | √ | √ |
